# Associations of dietary patterns with risk of incident atrial fibrillation in the REasons for Geographic And Racial Differences in Stroke (REGARDS)

**DOI:** 10.1007/s00394-023-03159-z

**Published:** 2023-04-29

**Authors:** Parveen K. Garg, Nicole Wilson, Emily B. Levitan, James M. Shikany, Virginia J. Howard, P. K. Newby, Suzanne Judd, George Howard, Mary Cushman, Elsayed Z. Soliman

**Affiliations:** 1 Division of Cardiology, USC Keck School of Medicine, 1510 San Pablo St. Suite 322, Los Angeles, CA 90033 USA; 2 Department of Biostatistics, University of Alabama at Birmingham, Birmingham, AL USA; 3 Department of Epidemiology, University of Alabama at Birmingham, Birmingham, AL USA; 4 Division of Preventive Medicine, School of Medicine, University of Alabama at Birmingham, Birmingham, AL USA; 5 Food Matters Media, LLC, Boston, MA USA; 6 Departments of Medicine and Pathology, Larner College of Medicine at the University of Vermont, Burlington, VT USA; 7 Department of Medicine, Epidemiological Cardiology Research Center (EPICARE), Section of Cardiovascular Medicine, Wake Forest School of Medicine, Winston-Salem, NC USA

**Keywords:** Atrial fibrillation, Mediterranean style diet, Dietary patterns, Prevention

## Abstract

**Background:**

We examined whether the risk of incident atrial fibrillation (AF) in a large, biracial, prospective cohort is lower in participants who adhere to heart-healthy dietary patterns and higher in participants who adhere to less heart-healthy diets.

**Methods:**

Between 2003 and 2007, the REasons for Geographic and Racial Differences in Stroke (REGARDS) cohort study enrolled 30,239 Black and White Americans aged 45 years or older. Dietary patterns (convenience, plant-based, sweets, Southern, and alcohol and salads) and the Mediterranean diet score (MDS) were derived based on food frequency questionnaire data. The primary outcome was incident AF at the follow-up visit 2013–2016, defined by either electrocardiogram or self-reported medical history of a physician diagnosis.

**Results:**

This study included 8977 participants (mean age 63 ± 8.3 years; 56% women; 30% Black) free of AF at baseline who completed the follow-up exam an average of 9.4 years later. A total of 782 incident AF cases were detected. In multivariable logistic regression analyses, neither the MDS score (odds ratio (OR) per SD increment = 1.03; 95% confidence interval (CI) 0.95–1.11) or the plant-based dietary pattern (OR per SD increment = 1.03; 95% CI 0.94–1.12) were associated with AF risk. Additionally, an increased AF risk was not associated with any of the less-healthy dietary patterns.

**Conclusions:**

While specific dietary patterns have been associated with AF risk factors, our findings fail to show an association between diet patterns and AF development.

**Supplementary Information:**

The online version contains supplementary material available at 10.1007/s00394-023-03159-z.

## Introduction

Atrial fibrillation (AF) is the most frequently encountered cardiac arrhythmia in clinical practice and more often seen in older populations. The overall prevalence of AF in the United States is 1% to 2% [1]. The prevalence of AF in European individuals over the age of 65 is estimated at 7.8% [2]. The presence of AF is associated with an over two-fold increased risk of stroke, heart failure, and cardiovascular mortality [3]. The risk of developing AF, however, is not evenly distributed amongst the various racial and ethnic groups. Despite underrepresented racial and ethnic groups experiencing a higher burden of traditional AF risk factors and a poorer AF-associated outcome profile, a paradox exists in that the overall incidence and prevalence of AF in these groups is only half of what is observed in White individuals [4, 5]. Continued investigations to improve our understanding of the mechanisms that underlie AF, as well as the differences in AF incidence observed across various racial and ethnic groups, are important.

Adherence to the Mediterranean or Dietary Approach to Stop Hypertension (DASH) diets are advocated by major medical societies due to their established effectiveness in reducing incident cardiovascular disease (CVD) [6–10]. The beneficial effects of diet on CVD risk reduction are thought to be due to reduction of inflammation and oxidative stress, mechanisms that are both implicated in the pathogenesis of AF [11–14]. A secondary analysis of the Prevención con Dieta Mediterránea (PREDIMED) trial found that individuals randomized to a Mediterranean diet, including the use of extra virgin olive oil (EVOO), experienced a substantially reduced risk of incident AF [15]. There is limited observational research, however, looking at associations of the Mediterranean diet and AF risk.

Similarly, prior study has also not evaluated whether the less-healthy Western dietary patterns that do not incorporate elements of a heart-healthy diet may place an individual at an increased risk for AF. Prior research in the REasons for Geographic And Racial Differences in Stroke (REGARDS) study demonstrated that a Southern dietary pattern, one that is high in added fats, fried food, eggs and egg dishes, organ meats, processed meats, and sugar-sweetened beverages, was associated with an increased risk of coronary heart disease and sudden cardiac death [16, 17]. We examined whether the risk of incident AF is lower in participants who adhere to heart-healthy dietary patterns—Mediterranean and plant-based—and higher in participants who adhere to less heart-health dietary patterns, e.g., the—Southern, sweets, or convenience as previously derived in REGARDS [18].

## Methods

Details of the methods of the REGARDS study have been published [19]. Briefly, REGARDS is a prospective cohort study designed to identify contributors to regional and Black-White disparities in stroke mortality. The study over-sampled Black persons and residents of the stroke belt (North Carolina, South Carolina, Georgia, Alabama, Mississippi, Tennessee, Arkansas, and Louisiana). Between January 2003 and October 2007, using postal mailings and telephone interviews, a total of 30,239 participants were recruited from a commercially available list of residents. Socio-demographic information and medical histories were obtained by a computer-assisted telephone interview (CATI). An in-home examination was performed 3–4 weeks after the telephone interview. Trained staff collected medication information, blood and urine samples, blood pressure readings, and a resting electrocardiogram (ECG). Approximately 10 years after the baseline assessment, 2013–2016, participants who were still alive and active completed a follow-up examination similar to the baseline visit. The institutional review boards at the collaborating centers approved the REGARDS study protocol, and all participants provided written informed consent.

### Dietary assessment

Diet was assessed at baseline with the Block 98 food frequency questionnaire (FFQ), a validated semi-quantitative FFQ that assessed usual dietary intake of 110 food items (NutritionQuest, Berkeley, CA) [19, 20]. For each line item on the FFQ, participants were asked how often, on average, they consumed the food (or group of foods) during the previous year, as well as the usual quantity of the food consumed. The FFQ also included adjustment questions (e.g., inquiring about the type of milk consumed—low-fat, non-fat, etc.) to further refine intake data. The FFQ was self-administered after the in-home visit and mailed to the REGARDS Operations Center, where they were reviewed for completeness and scanned. The results were then sent to NutritionQuest for scoring, which included a data set that provided the number of grams per day (g/day) for each line item on the FFQ. Amounts of each food on the FFQ consumed by a participant were calculated by multiplying the frequency of consumption of that food by the usual amount consumed. A total of 56 food groups, on which dietary patterns were based, were derived using the 110 individual food variables on the FFQ using published methods [18].

### Dietary patterns

Split sample replication was used to (1) derive the dietary patterns using exploratory factor analysis, and (2) test the patterns using confirmatory factor analysis [18, 21]. Patterns were named based on the major factor loadings. Factor 1 loaded heavily on mixed dishes, pasta dishes, pizza, Mexican food, and Chinese food and was designated the “Convenience” pattern. Factor 2 had high factor loadings for vegetables, fruits, fruit juice, cereal, beans, fish, poultry, and yogurt and was named the “Plant-based” pattern. Factor 3 loaded on added sugars, desserts, chocolate, candy, and sweetened breakfast foods and was named the “Sweets” pattern. Factor 4 loaded heavily on added fats, fried food, eggs and egg dishes, organ meats, processed meats, and sugar-sweetened beverages, reflecting a culinary pattern observed in the Southeastern United States, and was named the “Southern” pattern. Factor 5 loaded highly on beer, wine, liquor, green leafy vegetables, tomatoes, and salad dressing and was designated the “Alcohol and Salad” pattern. A standardized adherence score for each of the 5 dietary patterns was created (lower score = lower adherence).

Two patterns were considered to have health promoting properties based on their food composition and the general nutritional epidemiologic literature. Specifically, both the alcohol/salads and plant-based patterns had high factor loadings for foods such as vegetables, leafy greens, nuts, seeds, and fish, which are beneficial for health. We also identified three patterns, convenience, sweets, and southern, as unhealthy because they had higher factor loadings for foods that are known to be associated with unfavorable health and disease outcomes [18].

### Mediterranean diet score

The Mediterranean diet score (MDS) was derived according to published methods used in REGARDS based on the method of Trichopoulou and colleagues [22]. In brief, food group contributors to the MDS included those designated as “beneficial” (vegetables, fruits, legumes, cereals, fish), and “detrimental” (meat, dairy). One point was assigned for consumption that exceeded the sex-specific median for the “beneficial” groups or was below the median for “detrimental” food groups. For fat intake (eighth food category), participants with monounsaturated lipids to saturated lipids ratios at or above the sex-specific median were assigned a value of 1, and those with ratios below the sex-specific median were assigned a value of 0. For alcohol consumption (ninth category), participants were assigned a score of 1 for moderate consumption (> 0 and ≤ 7 drinks per week for women and > 0 and ≤ 14 drinks per week for men) and a score of 0 for everyone else. The MDS was determined by summing scores for the 9 food groups, resulting in a possible range of scores of 0 to 9.

### Atrial fibrillation

AF was identified at baseline and a subsequent follow-up visit approximately 10 years later by (1) a visit ECG and (2) self-reported history of a physician diagnosis during the CATI survey. The ECGs were read and coded at a central reading center (EPICARE, Wake Forest School of Medicine, Winston-Salem, NC) by analysts who were blinded to other REGARDS data. Self-reported AF was defined as an affirmative response to the following question: “Has a physician or a health professional ever told you that you had atrial fibrillation?” This question was as reliable a predictor of incident stroke events as AF detected by ECG [23].

### Covariates

Participant characteristics at baseline were used as covariates. Age, sex, race, household income, education, and smoking status were self-reported. Body mass index (BMI) and waist circumference were measured at the baseline examination. Physically active was defined as ≥ 4 days of exercise (enough to work up a sweat) per week. Hypertension was defined as systolic blood pressure ≥ 130 mm Hg, diastolic blood pressure ≥ 80 mm Hg, or self-reported current use of anti-hypertensive therapy. Dyslipidemia was defined as total cholesterol ≥ 240 mg/dL, low-density lipoprotein cholesterol ≥ 160 mg/dL, high-density lipoprotein cholesterol ≤ 40 mg/dL, or self-reported current use of lipid-lowering therapy. Diabetes mellitus was defined as fasting glucose ≥ 126 mg/ dL, non-fasting glucose ≥ 200 mg/dL, or self- reported current use of anti-diabetic medications. CRP measurement used a high-sensitivity particle-enhanced immunonephelometric assay on the BNIII nephelometer (N High Sensitivity CRP, Dade Behring Inc., Deerfield, IL) with an interassay coefficient of variation of 2–6%. CVD included the presence of coronary heart disease (a self-reported history of myocardial infarction, coronary artery bypass grafting, coronary angioplasty or stenting, or evidence of prior myocardial infarction on the baseline ECG) or prior stroke which was ascertained by participant’s self-report.

### Statistical analysis

Descriptive statistics for demographic, socioeconomic, lifestyle, anthropometric, and medical history variables at the baseline assessment according to quartiles of consumption of each dietary pattern and MDS categories were calculated using the chi-square test (for proportions) and analysis of variance (for continuous variables).

Logistic regression was used to calculate the odds ratio (OR) [95% confidence interval (CI)] for prevalent (baseline visit) and incident (follow-up visit) AF for each of the five dietary patterns as well as the MDS per standard deviation (SD) increment in adherence. Models were built as follows: model 1: age, sex, race, education, household income, and region; model 2: model 1 plus total energy intake, lifestyle factors (smoking, physical activity), CVD risk factors (BMI, waist circumference, hypertension, dyslipidemia, diabetes, history of CVD), and CRP. Statistical significance for all comparisons including interactions was defined as *p* < 0.05. SAS version 9.4 (Cary, NC) was used for all analyses.

Due to inherent limitations with the MDS, namely the designation of any dairy intake as adverse, and the availability of other scoring systems, we repeated the analyses using the Mediterranean-DASH Diet Intervention for Neurodegenerative Delay (MIND) diet score. Among the MIND diet components are 10 brain healthy food groups (green leafy vegetables, other vegetables, nuts, berries, beans, whole grains, seafood, poultry, olive oil and wine) and 5 unhealthy food groups (red meats, butter and stick margarine, cheese, pastries and sweets, and fried/fast food). This diet score has been associated with slower cognitive decline and reduced cardiovascular disease [24–26].

## Results

There were 18,967 REGARDS participants with complete covariate data and dietary assessment at baseline and 8,977 of these were included in the prospective analyses because they were without prevalent AF and completed a follow-up examination. Baseline characteristics stratified by MDS category and dietary pattern quartiles are shown in Table 1 and Supplemental Table 1, respectively. Mean (SD) age was 63 (8.3) years, 30% were Black, and 56% female. 782 (8.7%) participants developed AF over a median (IQR) follow-up of 9.3 (1.4) years. Compared to participants in the lowest MDS category, those in higher categories were older, more likely to be male, be black, have a high school education, be more physically active and less likely to smoke, have a low annual income, have hypertension, and have diabetes (Table 1).

**Table 1:** Baseline demographic characteristics by Mediterranean Diet Score category

Characteristic	Low adherence, *N* = 2607	Medium adherence, *N* = 3749	High adherence (score 6–9) *N* = 2503	*p*-trend across category
Age > 65	966 (37.1)	1545 (41.2)	1163 (46.5)	< 0.0001
Black	705 (27.0)	1136 (30.3)	804 (32.1)	< 0.0001
Male	1116 (42.8)	1603 (42.8)	1188 (47.5)	0.0009
Did not graduate HS	199 (7.6)	216 (5.8)	99 (4.0)	< 0.0001
Income < $20,000	346 (13.3)	389 (10.4)	191 (7.6)	< 0.0001
Residence in stroke belt	1504 (57.7)	2133 (56.9)	1304 (52.1)	0.0001
Current smoker	393 (15.1)	371 (9.9)	181 (7.2)	< 0.0001
Hypertension^*^	1377 (52.8)	1904 (50.8)	1246 (49.8)	0.03
Dyslipidemia^†^	1515 (58.1)	2109 (56.3)	1393 (55.7)	0.07
Diabetes^‡^	388 (14.9)	550 (14.7)	299 (11.9)	0.0027
Physically active^§^	709 (27.2)	1183 (31.6)	947 (37.8)	< 0.0001
Anti-hypertensive use	1185 (45.5)	1667 (44.5)	1076 (43.0)	0.04
Cardiovascular disease^‖^	298 (11.4)	409 (10.9)	299 (11.9)	0.58

^*^Hypertension defined as systolic blood pressure ≥ 130 mm Hg and/or diastolic blood pressure ≥ 80 mm Hg or self-reported current use of medication to control blood pressure

^†^Dyslipidemia defined as total cholesterol ≥ 240 mg/dL and/or low-density lipoprotein cholesterol ≥ 160 mg/dL and/or high-density lipoprotein cholesterol ≤ 40 mg/dL or self-reported current use of medication to control cholesterol

^‡^Diabetes mellitus defined as fasting glucose ≥ 126 mg/dL and/or non-fasting glucose ≥ 200 mg/dL or self-reported current use of medication to control blood sugar

^§^Physically active defined as ≥ 4 days of exercise (enough to work up a sweat) per week

^‖^Cardiovascular disease defined as the presence of coronary heart disease (a self-reported history of myocardial infarction, coronary artery bypass grafting, coronary angioplasty or stenting, or if evidence of prior myocardial infarction was present on the baseline ECG) or prior stroke which was ascertained by participant’s self-report

**Fig. 1:**
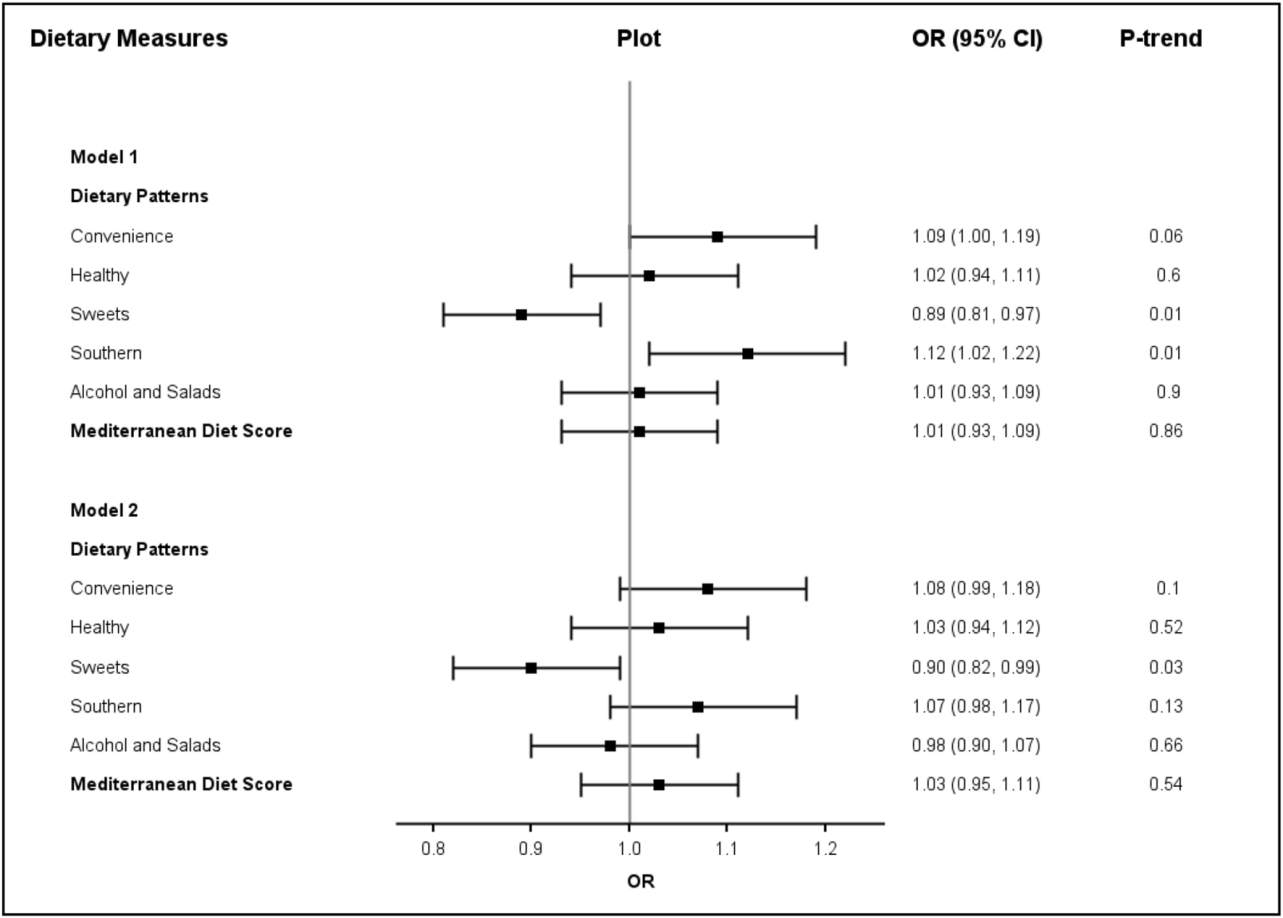
Risk of incident atrial fibrillation according to Dietary patterns and Mediterranean Diet Score. Data are presented as odds ratio (95% CI) per standard deviation increment. There were 782 incident AF cases among 8977 participants for dietary patterns analyses. There were 766 incident AF cases among 8859 participants for Mediterranean diet score analysis. *Model 1 adjusts for age, sex, race, education, household income, region, total energy. ^†^Model 2 adjusts for Model 1 + smoking, physical activity, body-mass index, waist circumference, hypertension, dyslipidemia, diabetes, cardiovascular disease, and C-reactive protein

Risk of incident AF according to the various dietary patterns and MDS are reported in Fig. 1. Neither the MDS score (OR per SD increment = 1.03; 95% CI 0.95–1.11) or the plant-based dietary pattern (OR per SD increment = 1.03; 95% CI 0.94–1.12) were associated with AF risk. In age-, race-, region-, sex and income-adjusted models, greater adherence to the sweets dietary pattern was associated with a lower AF risk (OR per SD increment = 0.89; 95% confidence interval (CI): 0.81, 0.97; p-trend = 0.01), while greater adherence to the Southern pattern was associated with an increased risk of AF (OR per SD increment = 1.12; 95% CI 1.02, 1.22; p-trend = 0.01) (Fig. 1). After additional adjustment for covariates, only the association between the sweets dietary pattern and AF remained significant (OR per SD increment = 0.90; 95% CI 0.82, 0.99; p-trend = 0.03).

Measures of adiposity and hypertension were most responsible for attenuation of association between Southern pattern and incident AF (Supplemental Table 2). The convenience and alcohol and salads dietary patterns were not associated with incident AF risk. In cross-sectional analyses, no associations were observed for any of the dietary patterns or MDS with prevalent AF (Supplemental Fig. 1). Findings were similar for both prevalent and incident AF when evaluating participants with the MIND diet score (Supplemental Table 3).

## Discussion

In a biracial cohort of individuals participating in the ongoing REGARDS study, a stronger adherence to a healthier dietary pattern, defined as either plant-based or Mediterranean, was not associated with a reduced incident AF risk. In addition, higher consumption of unhealthy Western dietary patterns, defined here as convenience, sweets, or Southern, was not associated with an increased incident AF risk.

Inflammation and oxidative stress are considered to play a role in AF pathogenesis and protective benefits of the Mediterranean diet are thought to involve modification of these pathways [12, 27, 28]. Anti-inflammatory and anti-oxidative properties are well documented in principal components of the Mediterranean diet [29–31]. A secondary analysis of the PREDIMED trial found that those randomized to a Mediterranean diet with EVOO experienced a 38% reduced risk of incident AF [15]. A similar association, however, was not found for participants in this trial randomized to the Mediterranean diet that included mixed nuts. No details were provided on the quantity of EVOO consumed or how much quantities differed amongst the EVOO, mixed nuts, and control groups, making it difficult to clinically interpret findings. Additional limitations of the PREDIMED trial included a sample size consisting of older White individuals at high cardiovascular risk and an unexpectedly low AF incidence (1.1% over 5 years) [15].

A “plant-based” dietary pattern is also thought to protect against AF development via similar mechanisms to those for the Mediterranean diet. Multiple large observational studies performed in biracial or multi-ethnic populations, however, reported no association between adherence to an American Heart Association recommended diet, which largely overlaps with a plant-based diet, and risk of AF [32–34]. Adherence in these studies were also measured using a FFQ, and diet scores were calculated as the sum of the scores for each of 5 individual components: fruits and vegetables (≥ 4.5 cups per day), fish (≥ 2 3.5-oz servings per week), fiber-rich whole grains (≥ 3 1-oz-equivalent serving per day), sodium (< 1500 mg/day), and sugar-sweetened beverages (≤ 450 kcal/week). The Framingham Heart Study, a predominantly white cohort of European ancestry, separately evaluated two of these components, fiber-rich whole grains and fish intake, and also reported no association [35].

The lack of association of MDS or plant-based dietary pattern with the risk of incident AF observed is consistent with much of the published observational literature mentioned above. Dietary factors do not appear to confer substantially to AF risk, and it is possible that any association with AF may be through indirect pathways where poor dietary habits increase the susceptibility to comorbidities that enhance an individual's risk for AF. It has been previously demonstrated that adherence to a plant-based diet reduces the likelihood of primary AF risk factors, including hypertension, obesity, diabetes, and coronary heart disease [36–41]. Blood pressure is reduced due to improved vasodilation and increased potassium intake [36, 42, 43]. Improvements in blood glucose control are thought to be due to the lower content of advanced glycation endproducts [44]. Higher fiber, lower fat content of plant‐based nutrition, and reduced energy density all may contribute to reduce obesity risk [39].

In this study, contrary to expectation, the sweets dietary pattern was independently associated with a reduction in AF risk. Although our findings run contrary to what we would have expected, a similar protective relationship was described in REGARDS for the sweets pattern with both sudden cardiac death in those with baseline coronary heart disease as well as incident stroke [16, 45]. It is well established, however, that a diet high in added sweets and saturated fats is associated with increased CVD incidence and cardiovascular mortality [46, 47].

A major limitation was that a reliable estimated energy requirement could not be calculated due to how physical activity was assessed, and adjustment for dietary under-reporting could not be performed, potentially introducing information bias. Additional limitations were also present. The majority of AF cases were by self-report at a single follow-up visit and participants may not have accurately recalled a diagnosis that occurred years earlier. More sensitive detection methods such as hospitalization diagnosis were not available. This likely led to the under ascertainment of AF events, especially those that were asymptomatic. This method of AF detection treated incident AF as a binary variable and did not allow use of longitudinal time to event analyses. The FFQ relied on recall of dietary intake, and this is prone to measurement error. Inaccuracies in reporting dietary intake may have resulted in misclassification, biasing results toward the null. Unfortunately, correction for potential measurement error was not possible. Diet was assessed at only one time point, so potential changes in diet could not be accounted for. Nearly 30% of participants did not complete a FFQ at baseline and these individuals were more likely to be Black race, less educated, and have a lower income. Finally, certain dietary pattern designations may not wholly depict each of the individual food components and it is possible that alternative naming options may be more representative; however, there are now multiple REGARDS publications using the names from the original design paper, so, for consistency, we have chosen to keep the names of the patterns the same.

In conclusion, in this large population-based sample of US adults, adherence to a variety of Western dietary patterns as well as to the Mediterranean diet were not associated with risk of incident AF. While specific dietary patterns have been strongly associated with AF risk factors, our findings suggest that they do not directly affect susceptibility to AF development.

## Supplementary Information

Below is the link to the electronic supplementary material.

Supplementary file1 (DOCX 72 KB)

## Data Availability

The data that support the findings of this study is available from the corresponding author upon reasonable request.

## References

[1] Go AS, Hylek EM, Phillips KA, Chang Y, Henault LE, Selby JV, Singer DE (2001). Prevalence of diagnosed atrial fibrillation in adults: national implications for rhythm management and stroke prevention: the AnTicoagulation and Risk Factors in Atrial Fibrillation (ATRIA) Study. JAMA.

[2] Di Carlo A, Bellino L, Consoli D, Mori F, Zaninelli A, Baldereschi M, Cattarinussi A, Alfonso MG, Gradia C (2019). Prevalence of atrial fibrillation in the Italian elderly population and projections from 2020 to 2060 for Italy and the European Union: the FAI Project. Europace.

[3] Odutayo A, Wong CX, Hsiao AJ, Hopewell S, Altman DG, Emdin CA (2016). Atrial fibrillation and risks of cardiovascular disease, renal disease, and death: systematic review and meta-analysis. BMJ.

[4] Borzecki AM, Keith Bridgers D, Liebschutz JM, Kader B, Lewis E, Kazis S, Berlowitz DR (2008). Racial differences in the prevalence of atrial fibrillation among males. J Natl Med Assoc.

[5] Rodriguez CJ, Soliman EZ, Alonso A, Swett K, Okin PM, Goff DC, Heckbert SR (2015) Atrial fibrillation incidence and risk factors in relation to race-ethnicity and the population attributable fraction of atrial fibrillation risk factors. the Multi-Ethnic Study of Atherosclerosis. Ann Epidemiol 25(2):71–76. 10.1016/j.annepidem.2014.11.024 10.1016/j.annepidem.2014.11.024 PMC4559265 25523897

[6] Estruch R, Ros E, Salas-Salvado J, Covas MI, Pharm D, Corella D, Aros F, Gomez-Garcia E, Ruiz-Gutierrez V (2013). Primary prevention of cardiovascular disease with a Mediterranean Diet. N Engl J Med.

[7] Fung TT, Rexrode KM, Mantzoros CS, Manson JE, Willett WC, Hu FB (2009). Mediterranean diet and incidence of and mortality from coronary heart disease and stroke in women. Circulation.

[8] Trichopoulou A, Costacou T, Bamia C, Trichopoulos D (2003). Adherence to a Mediterranean diet and survival in a Greek population. N Engl J Med.

[9] Levitan EB, Wolk A, Mittleman MA (2009). Consistency with the DASH diet and incidence of heart failure. Arch Intern Med.

[10] Fung TT, Chiuve SE, McCullough ML, Rexrode KM, Logroscino G, Hu FB (2008). Adherence to a DASH-style diet and risk of coronary heart disease and stroke in women. Arch Intern Med.

[11] Minich DM, Bland JS (2008). Dietary management of the metabolic syndrome beyond macronutrients. Nutr Rev.

[12] Aviles RJ, Martin DO, Apperson-Hansen C, Houghtaling PL, Rautaharju P, Kronmal RA, Tracy RP, Van Wagoner DR, Psaty BM, Lauer MS, Chung MK (2003). Inflammation as a risk factor for atrial fibrillation. Circulation.

[13] Liu T, Li G, Li L, Korantzopoulos P (2007). Association between C-reactive protein and recurrence of atrial fibrillation after successful electrical cardioversion: a meta-analysis. J Am Coll Cardiol.

[14] Harling L, Rasoli S, Vecht JA, Ashrafian H, Kourliouros A, Athanasiou T (2011). Do antioxidant vitamins have an anti-arrhythmic effect following cardiac surgery? A meta-analysis of randomised controlled trials. Heart.

[15] Martinez-Gonzalez MA, Toledo E, Aros F, Fiol M, Corella D, Salas-Salvado J, Rios E, Covas MI, Fernandez-Crehuet J (2014). Extravirgin olive oil consumption reduces risk of atrial fibrillation: the PREDIMED (Prevención con Dieta Mediterránea) trial. Circulation.

[16] Shikany JM, Safford MM, Soroka O, Brown TM, Newby PK, Durant RW, Judd SE (2021). Mediterranean diet score, dietary patterns, and risk of sudden cardiac death in the REGARDS study. J Am Heart Assoc.

[17] Shikany JM, Safford MM, Newby PK, Durant RW, Brown TM, Judd SE (2015). Southern dietary pattern is associated with hazard of acute coronary heart disease in the reasons for geographic and racial differences in stroke (REGARDS) study. Circulation.

[18] Judd SE, Letter AJ, Shikany JM, Roth DL, Newby PK (2015). Dietary patterns derived using exploratory and confirmatory factor analysis are stable and generalizable across race, region, and gender subgroups in the REGARDS study. Front Nutr.

[19] Howard VJ, Cushman M, Pulley L, Gomez CR, Go RC, Prineas RJ, Graham A, Moy CS, Howard G (2005). The reasons for geographic and racial differences in stroke study: objectives and design. Neuroepidemiology.

[20] Block G, Woods M, Potosky A, Clifford C (1990). Validation of a self- administered diet history questionnaire using multiple diet records. J Clin Epidemiol.

[21] Levine TR (2005). Confirmatory factor analysis and scale validation in communication research. Commun Res Rep.

[22] Tsivgoulis G, Judd S, Letter AJ, Alexandrov AV, Howard G, Nahab F, Unverzagt W, Moy C, Howard VJ, Kissela B, Wadley VG (2013). Adherence to a Mediterranean diet and risk of incident cognitive impairment. Neurology.

[23] Soliman EZ, Howard G, Meschia JF, Cushman M, Muntner P, Pullicino PM, McClure LA, Judd S, Howard VJ (2011). Self-reported atrial fibrillation and risk of stroke in the REasons for Geographic And Racial Differences in Stroke (REGARDS) study. Stroke.

[24] Morris MC, Tangney CC, Wang Y, Sacks FM, Barnes LL, Bennett DA, Aggarwal NT (2015). MIND diet slows cognitive decline with aging. Alzheimers Dement.

[25] Walker ME, O’Donnell AA, Himali JJ, Rajendran I, Melo van Lent D, Ataklte F, Jacques PF, Beiser AS, Seshadri S (2021). Approaches to stop hypertension intervention for neurodegenerative delay diet with cardiac remodeling in the community: the Framingham Heart Study. Br J Nutr.

[26] Golzarand M, Mirmiran P, Azizi F (2022). Adherence to the MIND diet and the risk of cardiovascular disease in adults: a cohort study. Food Funct.

[27] Guo Y, Lip GY, Apostolakis S (2012). Inflammation in atrial fibrillation. J Am Coll Cardiol.

[28] Youn JY, Zhang J, Zhang Y, Chen H, Liu D, Ping P, Weiss JN, Cai H (2013). Oxidative stress in atrial fibrillation: an emerging role of NADPH oxidase. J Mol Cell Cardiol.

[29] King DE, Egan BM, Geesey ME (2003). Relation of dietary fat and fiber to elevation of C-reactive protein. Am J Cardiol.

[30] Jacobs DR, Andersen LF, Blomhoff R (2007). Whole-grain consumption is associated with a reduced risk of noncardiovascular, noncancer death attributed to inflammatory diseases in the Iowa Women’s Health Study. Am J Clin Nutr.

[31] Duda MK, O’Shea KM, Tintinu A, Xu W, Khairallah RJ, Barrows BR, Chess DJ, Azimzadeh AM, Harris WS, Sharov VG, Sabbah HN, Stanley WC (2009). Fish oil, but not flaxseed oil, decreases inflammation and prevents pressure overload-induced cardiac dysfunction. Cardiovasc Res.

[32] Ogunmoroti O, Michos ED, Aronis KN, Salami AJ, Blankstein R, Virani SS, Spatz ES, Allen NB, Rana JS, Blumenthal RS, Veledar E, Szklo M, Blaha MJ, Nasir K (2018). Life's simple 7 and the risk of atrial fibrillation: the multi-ethnic study of atherosclerosis. Atherosclerosis.

[33] Garg P, O’Neal WT, Chen LY, Loehr LR, Sotoodehnia N, Soliman EZ, Alonso A (2018). American Heart Association’s life simple 7 and risk of atrial fibrillation in a population without known cardiovascular disease: The ARIC (Atherosclerosis Risk in Communities) Study. J Am Heart Assoc.

[34] Garg PK, O’Neal WT, Ogunsua A, Howard G, Soliman EZ, Cushman M (2018). Usefulness of the American Heart Association’s life simple 7 to predict the risk of atrial fibrillation (from the reasons for geographic and racial differences in stroke [REGARDS] Study). Am J Cardiol.

[35] Shen J, Johnson VM, Sullivan LM, Jacques PF, Magnani JW, Lubitz SA, Pandey S, Levy D, Vasan RS, Quatromoni PA, Junyent M, Ordovas JM, Benjamin EJ (2011). Dietary factors and incident atrial fibrillation: the Framingham Heart Study. Am J Clin Nutr.

[36] Alexander S, Ostfeld RJ, Allen K, Williams KA (2017) A plant‐based diet and hypertension. J Geriatr Cardiol 14(5):327–330. 10.11909/j.issn.1671-5411.2017.05.014 10.11909/j.issn.1671-5411.2017.05.014 PMC5466938 28630611

[37] Yokoyama Y, Nishimura K, Barnard ND, Takegami M, Watanabe M, Sekikawa A, Okamura T, Miyamoto Y (2014). Vegetarian diets and blood pressure: a meta-analysis. JAMA Intern Med.

[38] Yokoyama Y, Barnard ND, Levin SM, Watanabe M (2014). Vegetarian diets and glycemic control in diabetes: a systematic review and meta-analysis. Cardiovasc Diagn Ther.

[39] Barnard ND, Levin SM, Yokoyama Y (2015). A systematic review and meta- analysis of changes in body weight in clinical trials of vegetarian diets. J Acad Nutr Diet.

[40] Tonstad S, Butler T, Yan R, Fraser GE (2009). Type of vegetarian diet, body weight, and prevalence of type 2 diabetes. Diabetes Care.

[41] Ornish D, Scherwitz LW, Billings JH, Brown SE, Gould KL, Merritt TA, Sparler S, Armstrong WT, Ports TA, Kirkeeide RL, Hogeboom CH, Brand RJ (1998). Intensive lifestyle changes for reversal of coronary heart disease. JAMA.

[42] Cuspidi C, Tadic M, Grassi G (2019). How does blood pressure change in hypertensive patients with atrial fibrillation after successful electrical cardioversion?. J Clin Hypertens.

[43] Bazzano LA, Green T, Harrison TN, Reynolds K (2013). Dietary approaches to prevent hypertension. Curr Hypertens Rep.

[44] McMacken M, Shah S (2017) A plant‐based diet for the prevention and treatment of type 2 diabetes. J Geriatr Cardiol 14(5):342–354. 10.11909/j.issn.1671-5411.2017.05.009 10.11909/j.issn.1671-5411.2017.05.009 PMC5466941 28630614

[45] Sacks FM, Lichtenstein AH, Wu JH, Appel LJ, Creager MA, Kris-Etherton PM (2017). Dietary fats and cardiovascular disease: a presidential advisory from the American Heart Association. Circulation.

[46] Judd SE, Gutierrez OM, Newby PK, Howard G, Howard VJ, Locher JL, Kissela BM, Shikany JM (2013). Dietary patterns are associated with incident stroke and contribute to excess risk of stroke in Black Americans. Stroke.

[47] Yang Q, Zhang Z, Gregg EW, Flanders WD, Merritt R, Hu FB (2014). Added sugar intake and cardiovascular mortality among US adults. JAMA Intern Med.

