# Supplementary material for: Associations of dietary patterns with risk of incident atrial fibrillation in the REasons for Geographic And Racial Differences in Stroke (REGARDS)
Source: Eur J Nutr. 2023 Apr 29;62(6):2441–8. doi: 10.1007/s00394-023-03159-z (PMC10421757; doi:10.1007/s00394-023-03159-z)
Supplement: Supplementary file 1 — Supplementary file1 (DOCX 72 KB) [file 394_2023_3159_MOESM1_ESM.docx]

**Supplemental Table 1: Baseline characteristics according to dietary pattern quartile**

| Dietary Pattern |  | Quartile 1  (lowest adherence) | Quartile 2 | Quartile 3 | Quartile 4  (highest adherence) | *p*-trend across category |
| --- | --- | --- | --- | --- | --- | --- |
|  |  | N=1951 | N=2201 | N=2364 | N=2461 |  |
| Convenience | Age >65 | 1014 (52.0) | 999 (45.4) | 973 (41.2) | 742 (30.2) | <0.0001 |
|  | Black | 860 (44.1) | 771 (35.0) | 594 (25.1) | 469 (19.1) | <0.0001 |
|  | Male | 611 (31.3) | 850 (38.6) | 1128 (47.7) | 1371 (55.7) | <0.0001 |
|  | Did not graduate HS | 178 (9.1) | 142 (6.5) | 101 (4.3) | 103 (4.2) | <0.0001 |
|  | Income < $20,000 | 304 (15.6) | 239 (10.9) | 219 (9.3) | 179 (7.3) | <0.0001 |
|  | Residence in stroke belt | 1219 (62.5) | 1284 (58.3) | 1275 (53.9) | 1223 (49.7) | <0.0001 |
|  | Current smoker | 201 (10.3) | 243 (11.0) | 237 (10.0) | 275 (11.2) | 0.5756 |
|  | Hypertension^*^ | 1114 (57.1) | 1201 (54.6) | 1154 (48.8) | 1126 (45.8) | <0.0001 |
|  | Dyslipidemia^†^ | 1079 (55.3) | 1206 (54.8) | 1365 (57.7) | 1438 (58.4) | 0.0073 |
|  | Diabetes^‡^ | 325 (16.7) | 340 (15.4) | 278 (11.8) | 318 (12.9) | <0.0001 |
|  | Physically active^§^ | 679 (34.8) | 682 (31.0) | 746 (31.6) | 764 (31.0) | 0.0741 |
|  | Anti-hypertensive use | 969 (49.7) | 1047 (47.6) | 1004 (42.5) | 968 (39.3) | <0.0001 |
|  | Cardiovascular disease^‖^ | 230 (11.8) | 257 (11.7) | 287 (12.1) | 248 (10.1) | 0.0933 |
|  |  |  |  |  |  |  |
|  |  | N=2142 | N=2251 | N=2289 | N=2295 |  |
| Plant-based | Age >65 | 671 (31.3) | 936 (41.6) | 1059 (46.3) | 1062 (46.3) | <0.0001 |
|  | Black | 524 (24.5) | 675 (30.0) | 707 (30.9) | 788 (34.3) | <0.0001 |
|  | Male | 1186 (55.4) | 1028 (45.7) | 901 (39.4) | 845 (36.8) | <0.0001 |
|  | Did not graduate HS | 133 (6.2) | 150 (6.7) | 118 (5.2) | 123 (5.4) | 0.0647 |
|  | Income < $20,000 | 232 (10.8) | 240 (10.7) | 230 (10.0) | 239 (10.4) | 0.0186 |
|  | Residence in stroke belt | 1177 (54.9) | 1301 (57.8) | 1265 (55.3) | 1258 (54.8) | 0.3213 |
|  | Current smoker | 408 (19.0) | 261 (11.6) | 165 (7.2) | 122 (5.3) | <0.0001 |
|  | Hypertension^*^ | 1052 (49.1) | 1145 (50.9) | 1189 (51.9) | 1209 (52.7) | 0.0136 |
|  | Dyslipidemia^†^ | 1295 (60.5) | 1296 (57.6) | 1267 (55.4) | 1230 (53.6) | <0.0001 |
|  | Diabetes^‡^ | 263 (12.3) | 311 (13.8) | 326 (14.2) | 361 (15.7) | 0.0011 |
|  | Physically active^§^ | 561 (26.2) | 701 (31.1) | 733 (32.0) | 876 (38.2) | <0.0001 |
|  | Anti-hypertensive use | 883 (41.2) | 996 (44.2) | 1047 (45.7) | 1062 (46.3) | 0.0003 |
|  | Cardiovascular disease^‖^ | 239 (11.2) | 259 (11.5) | 266 (11.6) | 258 (11.2) | 0.9157 |
|  |  |  |  |  |  |  |
|  |  | N=2219 | N=2286 | N=2255 | N=2217 |  |
| Sweets | Age >65 | 877 (39.5) | 1002 (43.8) | 968 (42.9) | 881 (39.7) | 0.9526 |
|  | Black | 896 (40.4) | 685 (30.0) | 560 (24.8) | 553 (24.9) | <0.0001 |
|  | Male | 892 (40.2) | 946 (41.4) | 1044 (46.3) | 1078 (48.6) | <0.0001 |
|  | Did not graduate HS | 132 (5.9) | 130 (5.7) | 115 (5.1) | 147 (6.6) | 0.5190 |
|  | Income < $20,000 | 221 (10.0) | 216 (9.4) | 235 (10.4) | 269 (12.1) | 0.0252 |
|  | Residence in stroke belt | 1174 (52.9) | 1253 (54.8) | 1263 (56.0) | 1311 (59.1) | <0.0001 |
|  | Current smoker | 211 (9.5) | 204 (8.9) | 254 (11.3) | 287 (12.9) | <0.0001 |
|  | Hypertension^*^ | 1199 (54.0) | 1186 (51.9) | 1141 (50.6) | 1069 (48.2) | <0.0001 |
|  | Dyslipidemia^†^ | 1191 (53.7) | 1308 (57.2) | 1282 (56.9) | 1307 (59.0) | 0.0010 |
|  | Diabetes^‡^ | 327 (14.7) | 350 (15.3) | 317 (14.1) | 267 (12.0) | 0.0046 |
|  | Physically active^§^ | 736 (33.2) | 739 (32.3) | 710 (31.5) | 686 (30.9) | <0.0001 |
|  | Anti-hypertensive use | 1050 (47.3) | 1042 (45.6) | 972 (43.1) | 924 (41.7) | <0.0001 |
|  | Cardiovascular disease^‖^ | 253 (11.4) | 261 (11.4) | 257 (11.4) | 251 (11.3) | 0.9312 |
|  |  |  |  |  |  |  |
|  |  | N=2674 | N=2310 | N=2096 | N=1897 |  |
| Southern | Age >65 | 1132 (42.3) | 994 (43.0) | 893 (42.6) | 709 (37.4) | 0.0042 |
|  | Black | 225 (8.4) | 530 (22.9) | 828 (39.5) | 1111 (58.6) | <0.0001 |
|  | Male | 1014 (37.9) | 912 (39.5) | 998 (47.6) | 1036 (54.6) | <0.0001 |
|  | Did not graduate HS | 73 (2.7) | 93 (4.0) | 139 (6.6) | 219 (11.5) | <0.0001 |
|  | Income < $20,000 | 136 (5.1) | 196 (8.5) | 269 (12.8) | 340 (17.9) | <0.0001 |
|  | Residence in stroke belt | 1252 (46.8) | 1259 (54.5) | 1242 (59.3) | 1248 (65.8) | <0.0001 |
|  | Current smoker | 173 (6.5) | 202 (8.7) | 259 (12.4) | 322 (17.0) | <0.0001 |
|  | Hypertension^*^ | 1095 (40.9) | 1147 (49.7) | 1174 (56.0) | 1179 (62.2) | <0.0001 |
|  | Dyslipidemia^†^ | 1431 (53.5) | 1307 (56.6) | 1228 (58.6) | 1122 (59.1) | <0.0001 |
|  | Diabetes^‡^ | 215 ( 8.0) | 283 (12.3) | 344 (16.4) | 419 (22.1) | <0.0001 |
|  | Physically active^§^ | 914 (34.2) | 694 (30.0) | 655 (31.3) | 608 (32.1) | <0.0001 |
|  | Anti-hypertensive use | 950 (35.5) | 992 (42.9) | 1021 (48.7) | 1025 (54.0) | <0.0001 |
|  | Cardiovascular disease^‖^ | 291 (10.9) | 256 (11.1) | 265 (12.6) | 210 (11.1) | 0.3777 |
|  |  |  |  |  |  |  |
|  |  | N=2002 | N=2165 | N=2334 | N=2476 |  |
| Alcohol & Salads | Age >65 | 933 (46.6) | 926 (42.8) | 951 (40.7) | 918 (37.1) | <0.0001 |
|  | Black | 948 (47.4) | 768 (35.5) | 565 (24.2) | 413 (16.7) | <0.0001 |
|  | Male | 737 (36.8) | 859 (39.7) | 1082 (46.4) | 1282 (51.8) | <0.0001 |
|  | Did not graduate HS | 206 (10.3) | 146 (6.7) | 100 (4.3) | 72 (2.9) | <0.0001 |
|  | Income < $20,000 | 370 (18.5) | 247 (11.4) | 192 (8.2) | 132 (5.3) | <0.0001 |
|  | Residence in stroke belt | 1173 (58.6) | 1298 (60.0) | 1303 (55.8) | 1227 (49.6) | <0.0001 |
|  | Current smoker | 175 (8.7) | 233 (10.8) | 260 (11.1) | 288 (11.6) | 0.0029 |
|  | Hypertension^*^ | 1135 (56.7) | 1139 (52.6) | 1129 (48.4) | 1192 (48.1) | <0.0001 |
|  | Dyslipidemia^†^ | 1174 (58.6) | 1258 (58.1) | 1290 (55.3) | 1366 (55.2) | 0.0051 |
|  | Diabetes^‡^ | 310 (15.5) | 334 (15.4) | 331 (14.2) | 286 (11.6) | <0.0001 |
|  | Physically active^§^ | 614 (30.7) | 685 (31.6) | 725 (31.1) | 847 (34.2) | <0.0001 |
|  | Anti-hypertensive use | 991 (49.5) | 998 (46.1) | 986 (42.2) | 1013 (40.9) | <0.0001 |
|  | Cardiovascular disease^‖^ | 232 (11.6) | 252 (11.6) | 258 (11.1) | 280 (11.3) | 0.6463 |

Data are presented as frequency (percent)

*Hypertension defined as systolic blood pressure ≥130 mm Hg and/or diastolic blood pressure ≥80 mm Hg or self-reported current use of medication to control blood pressure.

†Dyslipidemia defined as total cholesterol ≥240 mg/dL and/or low-density lipoprotein cholesterol ≥160 mg/dL and/or high-density lipoprotein cholesterol ≤40 mg/dL or self-reported current use of medication to control cholesterol.

‡Diabetes mellitus defined as fasting glucose ≥126 mg/dL and/or non-fasting glucose ≥200 mg/dL or self-reported current use of medication to control blood sugar.

§Physically active defined as ≥4 days of exercise (enough to work up a sweat) per week.

‖Cardiovascular disease defined as the presence of coronary heart disease (a self-reported history of myocardial infarction, coronary artery bypass grafting, coronary angioplasty or stenting, or if evidence of prior myocardial infarction was present on the baseline ECG) or prior stroke which was ascertained by participant’s self-report.

**Supplemental Table 2: Attenuation (%) for individual variables in Model 2 per standard deviation increment in Southern Dietary Pattern adherence**

| Southern Dietary Pattern | HR (95%CI) | *Attenuation (%)* |
| --- | --- | --- |
| Model 1 (MOD1)* | 1.12 (1.02, 1.22) | --- |
| MOD1 + BMI | 1.08 (0.99, 1.18) | 26.92 |
| MOD1 + Waist | 1.07 (0.98, 1.17) | 35.65 |
| MOD1 + Smoking | 1.12 (1.03, 1.22) | -1.16 |
| MOD1 + Physical Activity | 1.12 (1.02, 1.22) | 0.31 |
| MOD1 + Hypertension | 1.10 (1.01, 1.20) | 16.27 |
| MOD1 + Dyslipidemia | 1.12 (1.02, 1.22) | 1.12 |
| MOD1 + Diabetes | 1.11 (1.02, 1.21) | 7.43 |
| MOD1 + Cardiovascular disease | 1.12 (1.03, 1.22) | -1.47 |
| MOD1 + CRP | 1.12 (1.02, 1.22) | 0.94 |

*Model 1 adjusts for age, sex, race, education, household income, region, total energy

**Supplemental Table 3:** **Risk of atrial fibrillation according to the MIND Diet Score**

|  | Standardized MIND Score  HR (95% CI) |
| --- | --- |
|  |  |
| Prevalent AF |  |
| #cases/total: 1599/18,967 |  |
| Model 1^*^ | 0.97 (0.91, 1.02) |
| Model 2^†^ | 0.98 (0.93, 1.04) |
|  |  |
| Incident AF |  |
| #cases/total: 782/8977 |  |
| Model 1^*^ | 1.00 (0.92, 1.08) |
| Model 2^†^ | 1.03 (0.95, 1.11) |

Data are presented as odds ratio (95% CI) per standard deviation increment

*Model 1 adjusts for age, sex, race, education, household income, region, total energy

†Model 2 adjusts for Model 1 + smoking, physical activity, body-mass index, waist circumference, hypertension, dyslipidemia, diabetes, cardiovascular disease, and C-reactive protein

**Supplemental Figure 1:** **Risk of prevalent atrial fibrillation according to Dietary patterns and Mediterranean Diet Score**


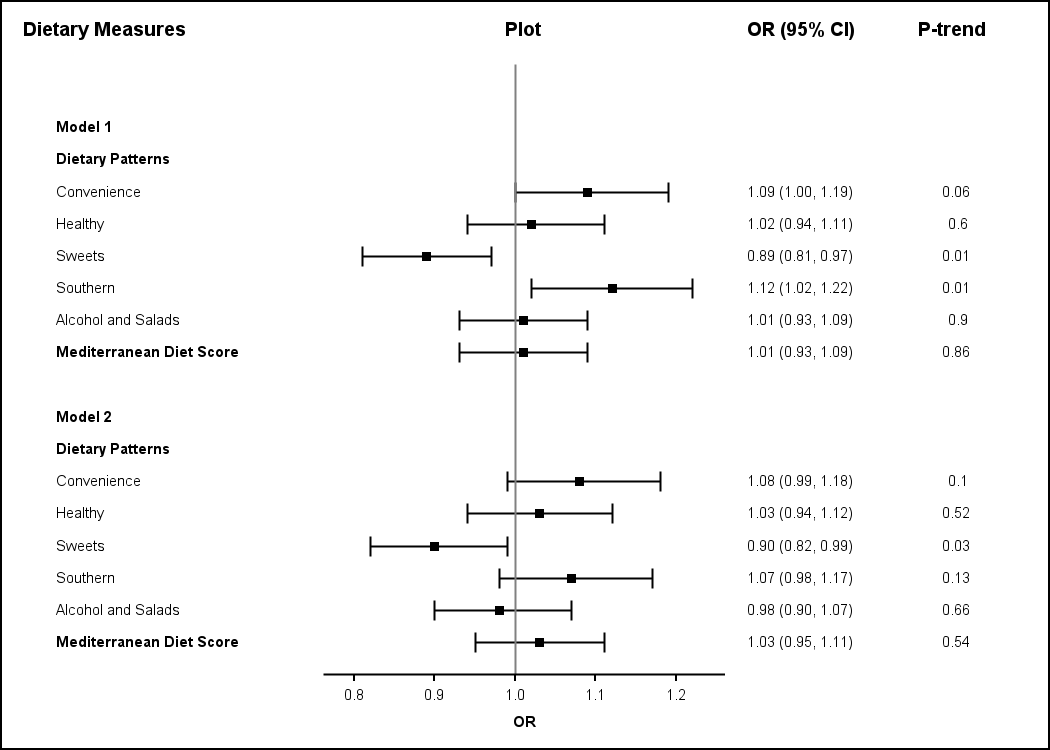


Data are presented as odds ratio (95% CI) per standard deviation increment. There were 1599 incident AF cases among 18967 participants for dietary patterns analyses. There were 1569 incident AF cases among 18661 participants for Mediterranean diet score analysis.

*Model 1 adjusts for age, sex, race, education, household income, region, total energy

†Model 2 adjusts for Model 1 + smoking, physical activity, body-mass index, waist circumference, hypertension, dyslipidemia, diabetes, cardiovascular disease, and C-reactive protein
